# Metagenomic Analyses Reveal That Energy Transfer Gene Abundances Can Predict the Syntrophic Potential of Environmental Microbial Communities

**DOI:** 10.3390/microorganisms4010005

**Published:** 2016-01-05

**Authors:** Lisa Oberding, Lisa M. Gieg

**Affiliations:** Department of Biological Sciences, Faculty of Science, University of Calgary, 2500 University Drive N.W., Calgary, AB T2N 1N4, Canada; l.k.oberding@gmail.com

**Keywords:** syntrophy, metagenomics, hydrocarbon biodegradation, principal component analysis, hydrocarbon metagenomics project, methanogenesis, microbial interactions

## Abstract

Hydrocarbon compounds can be biodegraded by anaerobic microorganisms to form methane through an energetically interdependent metabolic process known as syntrophy. The microorganisms that perform this process as well as the energy transfer mechanisms involved are difficult to study and thus are still poorly understood, especially on an environmental scale. Here, metagenomic data was analyzed for specific clusters of orthologous groups (COGs) related to key energy transfer genes thus far identified in syntrophic bacteria, and principal component analysis was used in order to determine whether potentially syntrophic environments could be distinguished using these syntroph related COGs as opposed to universally present COGs. We found that COGs related to hydrogenase and formate dehydrogenase genes were able to distinguish known syntrophic consortia and environments with the potential for syntrophy from non-syntrophic environments, indicating that these COGs could be used as a tool to identify syntrophic hydrocarbon biodegrading environments using metagenomic data.

## 1. Introduction

Many hydrocarbon compounds are both toxic to living organisms and difficult to remove from the environment. Aerobic microorganisms are capable of exploiting the reactive properties of oxygen to activate and biodegrade these compounds, and these mechanisms are well understood. However, petroleum contaminated environments quickly become anoxic [1,2]. In these environments, anaerobic electron acceptors such as nitrate (NO_3_^−^), iron (Fe^3+^), and sulfate (SO_4_^2−^) are rapidly utilized, leaving an environment with recalcitrant hydrocarbons which is depleted in electron acceptors [1]. Under these energy-limited methanogenic conditions, mechanisms of hydrocarbon biodegradation are poorly understood, though this is thought to be the main biodegradation process in contaminated environments [2,3]. 

Certain anaerobic bacteria (typically *Deltaproteobacteria* and *Firmicutes,* though other groups such as *Epsilonbacteria* may also play a role) are capable of breaking down hydrocarbon compounds under anoxic conditions, generating simple molecules such as hydrogen and acetate [4,5,6]. Though possible, these conversions are energetically unfavorable under standard conditions [3,5]. This thermodynamic barrier can be overcome through cooperation with other microorganisms such as methanogenic archaea that convert the simple molecules produced by the bacteria to methane and CO_2_ or H_2_O [5]. These latter reactions reduce the concentrations of these hydrocarbon breakdown byproducts, which drives the reaction kinetics forward and produces an overall reaction that is spontaneous [5]. Known as syntrophy (“feeding together”), such cooperation is essential for hydrocarbon biodegradation under methanogenic conditions, and produces just enough energy to sustain life; the energy gained by the entire syntrophic consortia is barely enough to synthesize one ATP [5,7].

Methanogenic syntrophy has been found across many environments and species. Wastewater treatment systems, bogs, ruminant digestive tracts, and landfills can all harbor syntrophic methanogenic communities that degrade a variety of substrates such as amino acids, aromatic and aliphatic hydrocarbons, and volatile fatty acids [5,6,8]. Methanogenic biodegradation of all hydrocarbon compounds relies on syntrophic interactions; however, much about the specific syntrophic mechanisms involved remains unknown as these communities and microorganisms are often difficult to culture and study [8]. What is known is that in petroleum-contaminated environments, these syntrophic activities are of high importance both ecologically as well as industrially, as they are key in both environmental bioremediation and in the transformation of high quality light oil into heavy crude in reservoirs [3,9].

In order to better understand the microbes and metabolic processes involved in environments where hydrocarbons are present, a project known as the Hydrocarbon Metagenomics Project (www.hmp.ucalgary.ca) was conducted in order to sequence and examine metagenomes from hydrocarbon resource environments [9,10]. The outcome of this project has led to a database containing multiple metagenomes that have been sequenced from various hydrocarbon laden environments, including aerobic and anaerobic waters and soils, oil reservoirs and coal seams, oil sands tailings ponds, drilling cores, and known syntrophic hydrocarbon-biodegrading laboratory consortia, allowing researchers to examine the diversity of genes and species present in these complex, difficult to culture microbial ecosystems [9].

Energy transfer mechanisms that occur during the syntrophic metabolism of any compound are complex, and there is still much to understand about how energetic coordination occurs. Though direct interspecies electron transfer (DIET) can be involved in syntrophic metabolism involving iron-reducing bacteria [8,11], electron transfer through hydrogen and formate is thought to be a primary mechanism used by most organisms capable of syntrophy (herein referred to as “syntrophs” or “syntrophic organisms”) by which energy is transferred during syntrophic processes [8]. The genomes of pure syntroph strains and their methanogenic partners have been found to harbor multiple formate dehydrogenase and hydrogenase genes [8]. These can be involved in electron confurcation, coupling the thermodynamically favorable oxidation of ferredoxin to drive the normally unfavorable production of formate and hydrogen from NADH. Hydrogenases and formate dehydrogenases can also be membrane associated, potentially using ion gradients to drive reverse electron transfer [8,12]. Membrane associated FeS oxidoreductases present in syntrophic bacteria could be involved in funneling electrons to redox carriers to facilitate these reactions [8]. Fix proteins, which are membrane-bound electron transfer flavoprotein:quinone oxidoreductases, are proposed to utilize the energy in the ion gradient to supply reduced ferreredoxin needed for syntrophic metabolism [8,13]. Fnr proteins, which are ion translocating ferredoxin:NAD^+^ oxidoreductases, may also function in reverse electron transfer by using the ion gradient to drive the unfavorable reduction of ferredoxin with NADH [8,14]. 

We sought to determine whether environments with the potential for syntrophic hydrocarbon biodegradation to be the dominant microbial lifestyle could be identified through analysis of metagenomic data. We hypothesized that gene families associated with syntrophic energy transfer previously identified in the genomes of pure syntrophic strains [8] could be used to distinguish syntrophic from non syntrophic communities. In order to do this, metagenomes were classified based on their potential to have syntrophic processes as a dominant microbial lifestyle according to their sampling location information and microbial community composition. Clusters of orthologous groups (COGs) associated with various categories of genes found in syntroph genomes and known to be involved in syntrophic energy transfer were determined using the Joint Genome Institute’s Integrated Microbial Genomes Database [8,15]. Detection of these COGs in metagenomes from both hydrocarbon-related and non-hydrocarbon related environments was performed utilizing the Joint Genome Institute’s Integrated Metagenomes Database [16]. The results of this analysis were then compared using principal component analysis to determine if environments with syntrophic potential would cluster together with the metagenomes from known syntrophic cultures. Analysis of universally present COGs was also performed alongside the syntroph associated COGs in order to determine if the syntroph associated COGs collectively could distinguish syntrophic communities [17].

## 2. Materials and Methods

### 2.1. Selection of Metagenomes

Metagenomes from hydrocarbon resource environments were obtained from the Hydrocarbon Metagenomics Project (HMP) website, and accessed through the Joint Genome Institute’s Integrated Metagenomes database (IMG) [10,16]. Other metagenomes were obtained by searching the IMG database for publicly released metagenome datasets from a variety of environments [16]. Information for each metagenome analyzed in this study along with an associated identification number is listed in Table 1. Metagenomes were accessed in the IMG database in November of 2015.

**Table 1:** Metagenomes analyzed in this study. All metagenomes were publically available on the IMG database. Source indicates whether metagenome was originally obtained through the Hydrocarbon Metagenomics Project (HMP) or through searching the IMG database [9,10,16]. Classification was performed according to sampling location information as well as microbial community composition (Figure 1). **^†^** Community information not available.

#	Metagenome Name	IMG Genome ID	Source	Gene Count	Classification
1	Marine microbial communities from Deepwater Horizon subsurface plume in Gulf of Mexico, 16-4 Below Plume (16-4 Below Plume)	3300005379	IMG	112790	Non-Syntrophic Hydrocarbon
2	Marine microbial communities from Deepwater Horizon subsurface plume in Gulf of Mexico, 16-5 In Plume (16-5 In Plume)	3300005380	IMG	114085	Non-Syntrophic Hydrocarbon
3	Marine microbial communities from Deepwater Horizon subsurface plume in Gulf of Mexico, 52-1 Below Plume (52-1 Below Plume)	2149837027	IMG	60113	Non-Syntrophic Hydrocarbon
4	Marine microbial communities from Deepwater Horizon subsurface plume in Gulf of Mexico, 52-4 In plume (52-4 In Plume)	3300005378	IMG	102800	Non-Syntrophic Hydrocarbon
5	Oil sands microbial communities from Horse River, Alberta, Canada—outcrops (H1C: 454 sequencing assembly)	3300001422	HMP	570427	Non-Syntrophic Hydrocarbon
6	Oil sands microbial communities from Horse River, Alberta, Canada—outcrops collected from inside the river (H1R: 454 sequencing assembly)	3300001393	HMP	559882	Non-Syntrophic Hydrocarbon
7 **^†^**	Syncrude MLSB Tailings Pond Water Surface—Tailings pond microbial communities from Northern Alberta—Syncrude Mildred Lake Settling Basin (PDSYNTPWS: 454+illumina sequencing assembly)	3300001605	HMP	3740874	Non-Syntrophic Hydrocarbon
8	Syncrude MLSB WIP Surface + Isolates—Tailings pond microbial communities from Northern Alberta—Syncrude Mildred Lake Settling Basin (WIP-PD_SYN_TP_WS_002_003_071511 and isolates PD8, PD9 joint assembly)	3300001239	HMP	768174	Non-Syntrophic Hydrocarbon
9	Wastewater microbial communities from Syncrude, Ft. McMurray, Alberta—Microbes from Oil-contaminated ecosystem in Alberta, Canada Inniskillen 604.3 (Inniskillen 604.3: 454 sequencing assembly)	3300001190	HMP	25694	Non-Syntrophic Hydrocarbon
10	Wastewater microbial communities from Syncrude, Ft. McMurray, Alberta—Microbes from Sediment core from a heavy oil reservoir, Alberta Canada Inniskillen 614.3 (Inniskillen 614.3: 454+illumina sequencing assembly)	3300001197	HMP	204944	Non-Syntrophic Hydrocarbon
11	Arctic peat soil from Barrow, Alaska—NGEE Surface sample 210-1 shallow-072012 (NGEE Surface sample 210-1 shallow-072012, ASSEMBLY_DATE=20130514)	3300001414	IMG	14379538	Non-Syntrophic
12	Arctic peat soil from Barrow, Alaska—NGEE Surface sample 210-2 deep-072012 (NGEE Surface sample 210-2 deep-072012, ASSEMBLY_DATE=20130514)	3300001396	IMG	8697097	Non-Syntrophic
13	Arctic peat soil from Barrow, Alaska—NGEE Surface sample 210-2 deep-092012 (NGEE Surface sample 210-2 deep-092012, ASSEMBLY_DATE=20130516)	3300001385	IMG	6241552	Non-Syntrophic
14	Arctic peat soil from Barrow, Alaska—NGEE Surface sample 210-2 shallow-072012 (NGEE Surface sample 210-2 shallow-072012, ASSEMBLY_DATE=20130514)	3300001416	IMG	14687361	Non-Syntrophic
15	Freshwater microbial communities from Lake Mendota, WI—02JUN2012 deep hole epilimnion (Lake Mendota Deep Hole Epilimnion 02Jun12, ASSEMBLY_DATE=20140125)	3300002296	IMG	1990049	Non-Syntrophic
16	Human retroauricular crease microbial communities from NIH, USA—visit 1, subject 338793263	7000000458	IMG	36795	Non-Syntrophic
17	Human right retroauricular crease microbial communities from NIH, USA—visit 2 of subject 763961826 replicate 2	7000000031	IMG	39970	Non-Syntrophic
18	Marine microbial communities from expanding oxygen minimum zones in Line P, North Pacific Ocean—August 2009 P16 10m (Line P August 2009 P16 10m, March 2012 Assem)	3300000149	IMG	238270	Non-Syntrophic
19	Marine microbial communities from expanding oxygen minimum zones in Line P, North Pacific Ocean—June 2008 P4 1300m (Line P June 2008 P4 1300m, March 2012 Assem)	3300000141	IMG	256292	Non-Syntrophic
20	Marine microbial communities from expanding oxygen minimum zones in the Saanich Inlet—54 02/08/11 120m (Saanich Inlet 54 02/08/11 120m, March 2012 Assem)	3300000146	IMG	169407	Non-Syntrophic
21	Soil microbial communities from Great Prairies—Iowa, Native Prairie soil (Iowa, Native Prairie soil, Feb 2012 Assem MSU hiseq + gaii)	3300000364	IMG	8508638	Non-Syntrophic
22	Subsurface groundwater microbial communities from S. Glens Falls, New York, USA—GMW60B uncontaminated upgradient, 5.4 m (Subsurface groundwater monitoring well GMW60B uncontam upgr,5.4m, Oct 2012 Assem)	3300000571	IMG	1391570	Non-Syntrophic
23	Switchgrass and industrial compost incubating bioreactor microbial communities from the Joint BioEnergy Institute, California, USA, that is aerobic and thermophilic—SG0.5JP960 (454-Illumina assembly) —version 2 (454-Illumina assembly v2)	3300005442	IMG	62968	Non-Syntrophic
24	Wastewater treatment Type I Accumulibacter community from EBPR Bioreactor in Madison, WI—N_134min_Aerobic	3300002344	IMG	367402	Non-Syntrophic
25	Coal bed methane well microbial communities from Alberta, Canada (CO182: coal bed cutting Illumina Assembly)	3300000052	HMP	665055	Other
26	Coal-degrading lab enrichment microbial communities from Bowden, Alberta, Canada—QSAFCN5 (QSAFCN5: 454 assembly)	3300000507	HMP	257387	Other
27	Coal-degrading lab enrichment microbial communities from Bowden, Alberta, Canada—methanogenic culture: QSAFCN2 (QSAFCN2 454 assembly)	3300000408	HMP	223117	Other
43	Sheep rumen microbial communities from New Zealand—Rank43_high (high_rank43)	3300001531	IMG	911348	Other
28	Benzene-degrading bioreactor microbial communities from Toronto, Ontario, Canada, that are methanogenic—September 2009 gDNA_4 (Assembly with PE data)	2061766000	IMG	207753	Syntrophic Culture
29	Hydrocarbon resource environments microbial communities from Canada and USA—Toluene degrading community from Alberta, Canada (Toluene: 454+illumina+illuminaFosmid sequencing assembly)	3300001567	HMP	1184637	Syntrophic Culture
30	Oil sands microbial community from Northern Alberta which degrade Naphthaline (NapDC: 454 and illumina hybrid assembly)	3300000032	HMP	749231	Syntrophic Culture
31	Tailings pond microbial communities from Northern Alberta—Short chain hydrocarbon degrading methanogenic enrichment culture SCADC: (SCADC: 454+illumina assembly)	3300000568	HMP	1513645	Syntrophic Culture
32	Wastewater bioreactor microbial communities from Singapore—TA reactor DNA contigs from 4 sample (re-annotation) (MER-FS) (assembled)	3300005443	IMG	95700	Syntrophic Culture
33	Subsurface groundwater microbial communities from S. Glens Falls, New York, USA—GMW46 contaminated, 5.4 m (Subsurface groundwater monitoring well GMW46 contaminated, 5.4m, Oct 2012 Assem)	3300000574	IMG	1250031	Potentially Syntrophic Hydrocarbon
34	Tailings pond microbial communities from Northern Alberta—TP6_2008_30ft:	2228664008	HMP	389458	Potentially Syntrophic Hydrocarbon
35	Tar lake microbial communities from La Brea, Trinidad and Tobago	2228664012	HMP	195772	Potentially Syntrophic Hydrocarbon
36	Wastewater microbial communities from Syncrude, Ft. McMurray, Alberta—Microbes from Suncor taillings pond 6 2012TP6_6 (2012TP6_6m: illumina sequencing assembly)	3300001580	HMP	3205687	Potentially Syntrophic Hydrocarbon
37	Wastewater microbial communities from Syncrude, Ft. McMurray, Alberta—Microbes in water sample from Medicine Hat oil field -PW_MHGC_2012April2: (PW_MHGC_2012April2: 454+illumina sequencing assembly final)	3300001444	HMP	2443755	Potentially Syntrophic Hydrocarbon
38	Wastewater microbial communities from Syncrude, Ft. McMurray, Alberta—Microbes in water sample from Medicine Hat oil field -PW_MHGC_2012April2: (PW_MHGC_2012April2: 454 + illumina sequencing assembly)	3300001592	HMP	2251644	Potentially Syntrophic Hydrocarbon
39	Tailings Pipe from MSLB 2011 -Wastewater microbial communities from Syncrude, Ft. McMurray, Alberta—West In Pit SyncrudeMLSB2011 (SyncrudeMLSB2011: 454+illumina assembly)	3300000558	HMP	3021632	Potentially Syntrophic Hydrocarbon
42	Marine sediment microbial communities from Arctic Ocean, off the coast from Alaska—sample from high methane PC12-225-485cm (High methane PC12-225-485cm Jan 2011 assembly)	2140918005	IMG	674403	Potentially Syntrophic

### 2.2. Metagenome Community Composition and Classification

Microbial community composition pertaining to the metagenomes obtained from the HMP was determined by using the 16s rRNA gene predictions from the 454 single end data available on the HMP database website for each metagenome [10]. Microbial community composition pertaining to the metagenomes obtained directly from the IMG database was inferred through the percent BLAST identities of the genes identified in the metagenome, with the settings set to identity +30% in order to decrease the proportion of “unclassified” community members [16]. From the total microbial community composition, only microbial groups with majority representation or those with relevance to the syntrophic potential of the environment were chosen for display. Metagenomes were then classified into categories of syntrophic potential based on their community composition as well as the environment from which the metagenome was obtained, as stated in the HMP and IMG databases [10,16].

### 2.3. Selection of Clusters of Orthologous Groups (COGs)

Clusters of orthologous groups (COGs) used for analysis were divided into two divisions—those that are universally present and those that are associated with genes present in syntroph genomes known to be involved in syntrophic energy transfer [8] (Table 2).

Universally present COGs were obtained from literature, and grouped into 4 categories as shown in Table 2 [17]. Genes found in various pure syntrophic strains that are related to syntrophic energy transfer mechanisms have been previously identified and were used as the predictive syntrophy genes in this study [8]. These genes were classified into categories based on their function (Table 2). Genes identified as being associated with DIET processes were excluded from this analysis because very few COGs were identified as being annotated for these genes, and the COGs that were annotated were found to be too general in their classification to be associated primarily with syntrophic processes (e.g., genes for flagella, pili, and cytochromes). In order to obtain clusters of orthologous groups (COGs) which are associated with these syntrophic processes, each gene listed by Sieber *et al.* [8] was examined using the IMG database, and all the COGs associated with each gene were obtained [15]. The COGs identified for genes belonging to the same category were then grouped, and any multiples were removed. The individual COGs belonging to each category are listed in Supplemental Tables S1 and S2.

**Table 2:** Categories of clusters of orthologous groups (COGs) searched in the metagenomes. Universally present COGs were obtained from previously published information [17]. Syntroph associated COG categories were obtained from the annotations in the IMG database for each of the genes for each respective category from previously published information [8,15]. Further information on the specific COGs searched in each category can be found in Supplemental Tables S1 and S2.

Universally Present COG Categories	G1-Ribosome and Translation Initiation
	G2-Ribosome Associated/ Protein Modification
	G3-Transcription/DNA Replication
	G4-Unknown
Syntroph Associated COG Categories	FeS Oxidoreductases
	Fnr
	Fix
	Confurcating Hydrogenases
	Other Hydrogenases
	Membrane Hydrogenases
	NADH Linked Formate Dehydrogenases
	Other Formate Dehydrogenases
	Membrane Formate Dehydrogenases

### 2.4. Principal Component Analysis

The number of each of the universally present and syntroph associated COGs found in each metagenome was determined using the IMG database (which utilizes the 2014 COG database) by viewing the functions *vs.* genomes in the function analysis profile and alignment tool in the combined assembled and unassembled metagenomic data [16]. The total numbers of each COG detected in each metagenome were then summed for each category as listed in Table 2. This number was then divided by the number of COGs represented by each category, before then being divided by the total number of genes detected in each of the metagenomes (using assembled and unassembled data) (Table 1) in order to normalize the total against the metagenome size. Metagenomes were all searched against the COG database in November of 2015.

The normalized total number of COGs for each category found in each metagenome was then used for principal component analysis using R [18]. Principal component analysis was performed on two separate datasets - one with the normalized total COGs per metagenome from each category of the universally present groups, and one with the normalized total COGs per metagenome from each category in both the universally present and the syntroph associated groups. Analysis was performed using the prcomp() function built into the R interface, with scaling set to “TRUE” in order to perform the analysis using a correlation matrix which normalizes the data by standardizing the variance in the data to one [18]. For each principal component analysis, a scree plot showing the amount of variance captured by each of the calculated principal components was generated alongside the statistics of the principal components to verify that the amount of variation encompassed by the first 2 principal components would sufficiently represent the major sources of variation in the data (Supplemental Figure S1 and Table S3 for analysis of universal COGs; Figure S2 and Table S4 for the analysis of both universal and syntroph associated COGs). Plotting of the resulting principal component analysis plot was performed using library(ggplot2) [19], combined with the stat_ellipse() function [20] to draw 95% confidence intervals around the user-defined “Syntrophic” and “Non-Syntrophic” groups (requires library(devtools) and library(digest) [21,22]). A circle of correlations plot was also drawn for each of the two datasets in order to better visualize the contribution of the different COG categories to the principal components.

The commented R scripts used in order to perform the analyses in this study are included in the supplemental data, including code to install the required packages. Additionally, the above analysis was repeated using KO (KEGG Orthology) categories detected for each of the syntroph-associated categories, as well as with universally present KOs, in order to determine whether similar results would be obtained using different databases (Table S5).

### 2.5. Analysis of COGs in Metagenomes

The total normalized number of COGs in each metagenome found for each of the syntroph associated COG groups was displayed on a surface plot in order to visualize the differences in COG number for each of the COG categories between metagenomes in the syntrophic group *versus* those in the non-syntrophic group. In addition, mean numbers of COGs between syntrophic and non-syntrophic metagenomes in each syntroph-associated category were examined, and t-tests were performed to determine if the mean number of COGs found in each category of syntroph associated COGs was statistically different between the syntrophic and non-syntrophic metagenomes.

## 3. Results

### 3.1. Metagenome Selection and Classification

In order to determine the syntrophic potential of hydrocarbon resource environments based on examining syntroph associated gene families in metagenomes, metagenomes were obtained which were sequenced and publically available on both the HMP website and the IMG database [10,16]. These included metagenomes that were sequenced from known syntrophic hydrocarbon-degrading consortia (#31—short chain alkanes, #29—toluene, and #30—naphtha), and metagenomes from various other aerobic and anaerobic environments where hydrocarbons are present (Table 1) [9].

In order to expand the dataset to include metagenomes from outside of the HMP, metagenomes sequenced from a variety of different environments were selected from the IMG database (Table 1) [16]. These included known syntrophic benzene (#28) and terephthalate-degrading (#32) mixed cultures, methanogenic marine sediments (#42), as well as a variety of aerobic and anaerobic environments and mixed cultures from non-hydrocarbon associated conditions.

**Figure 1:**
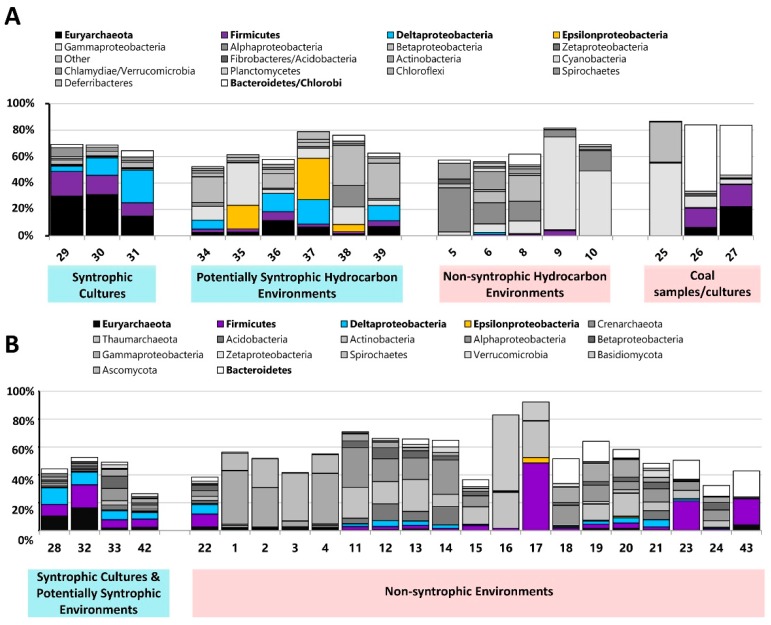
(**A**) Microbial community composition of metagenomes sequenced as part of the Hydrocarbon Metagenomics Project (HMP) [10]. Relevant community members of importance to this study are colored; all others are grey. Community composition was determined using the HMP database 454 single end data 16S rRNA based prediction (total community composition not shown) [9]. Based on sample location as well as microbial community, samples were grouped into categories for use in further analysis. (**B**) Microbial community composition of metagenomes obtained from the IMG database using the distribution by BLAST percent identities (cumulative) with percent hits 30%+ (total community composition not shown) [16]. Based on sample location as well as microbial community, samples were grouped into categories for use in further analysis.

We then determined the microbial community compositions of the metagenomes, and the metagenomes were classified into groups based on their sampling location information and their microbial community composition (Figure 1 and Table 1). Metagenomes from known syntrophic laboratory cultures were classified as “Syntrophic Cultures” to be used as a reference point for syntrophy in the principal component analysis. Hydrocarbon resource environments containing microbial communities typically associated with syntrophic processes (*Deltaproteobacteria*, *Firmicutes*, and *Epsilonproteobacteria* together with *Euryarchaeota*, primarily consisting of methanogens) and sampled from locations where anaerobic, methanogenic conditions dominate were classified as “Potentially Syntrophic Hydrocarbon Environments” (Figure 1A) [4,5,6]. Hydrocarbon environments that did not display this community signature and/or were sampled from conditions where other electron acceptors would be present were classified as “Non-Syntrophic Hydrocarbon Environments”. Three metagenomes from the HMP were classified into their own group (“Other”). Metagenome #25 was annotated as having been sampled from a coal bed methane sample (where syntrophic processes would presumably occur); however the microbial community composition indicated that no methanogens or known potential syntrophs were present in appreciable abundance [9,23]. Metagenomes #26 and #27 were obtained from a methanogenic coal-degrading culture, but sampling was performed very early in the cultures incubation, and no *Deltaproteobacteria* were found in the community profile [9,10]. As the community profile for these two metagenomes differed from the other known syntrophic cultures where *Deltaproteobacteria* were detected, and the COGs selected for this study were obtained primarily from *Deltaproteobacteria* genes, these metagenomes were classified separately from the other known syntrophic cultures (Figure 1A).

Metagenomes obtained directly from the IMG database were classified in a similar manner (Figure 1B). Metagenomes known to be from syntrophic cultures were classified as “Syntrophic Cultures”, two environments (#33 and #42) was classified as “Potentially Syntrophic”, and all other environments were classified as “Non-Syntrophic” primarily based on the lack of a microbial community which could act to syntrophically generate methane. The only exception to this was the sheep rumen metagenome (#43) which, as it showed a similar community profile to the coal-degrading cultures, was classified as “Other”. Certain metagenomes with potentially syntrophic community compositions were classified into this group based on details about their environment. Metagenome #22 was sampled from an uncontaminated location in the aquifer that #33 (contaminated) was obtained from, and as the influx of carbon (present in #33) which could be degraded syntrophically would not be present in this sample, syntrophy would likely not be the dominant microbial lifestyle of the microorganisms present. Metagenomes #19 and #20 also contained microorganisms that may be associated with syntrophic processes, but were sampled from locations where alternative electron acceptors (O_2_, SO_4_^2−^) would be present, and therefore syntrophic processes would likely not dominate. 

### 3.2. Principal Component Analysis

Principal component analysis was performed in order to determine if environments with the potential to be syntrophic would have a similar profile of syntroph associated COGs as known syntrophic cultures, and to see if the profile of syntrophassociated COGs could be used to identify these metagenomes as being distinctly different than environments where syntrophy would not be expected as the dominant microbial lifestyle. Using R, the normalized sum total of COGs found in each metagenome for each category was analyzed (with centering and using a correlation matrix) and plotted.

#### 3.2.1. Principal Component Analysis of Universally Present COGs

**Figure 2:**
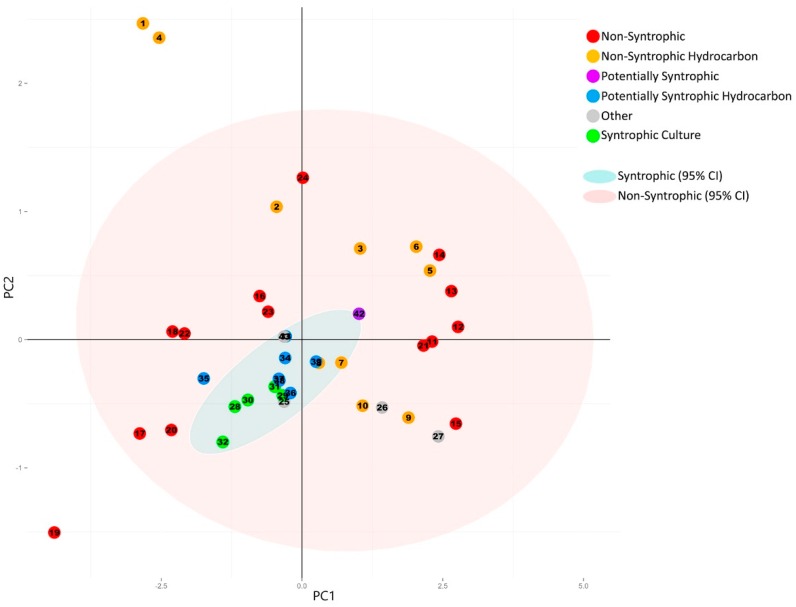
Principal component analysis plot generated from the normalized number of universally present COGs detected in each metagenome. Categories for the universally present COGs are listed in Table 2, with the individual COGs for each category listed in Supplemental Table S1. Numbers of individual COGs found in each metagenome were summed for each COG category, divided by the total number of COGs for each respective category, and the sum was normalized against the total number of genes detected in each metagenome (Table 1 and Table 2). Principal component analysis was performed using R [18]. 95% Confidence ellipses were drawn for all metagenomes classified as syntrophic/potentially syntrophic, and for all metagenomes classified as non-syntrophic. The first two principal components are shown. The corresponding statistics and scree plot are shown in Supplemental Figure S1 and Table S3.

**Figure 3:**
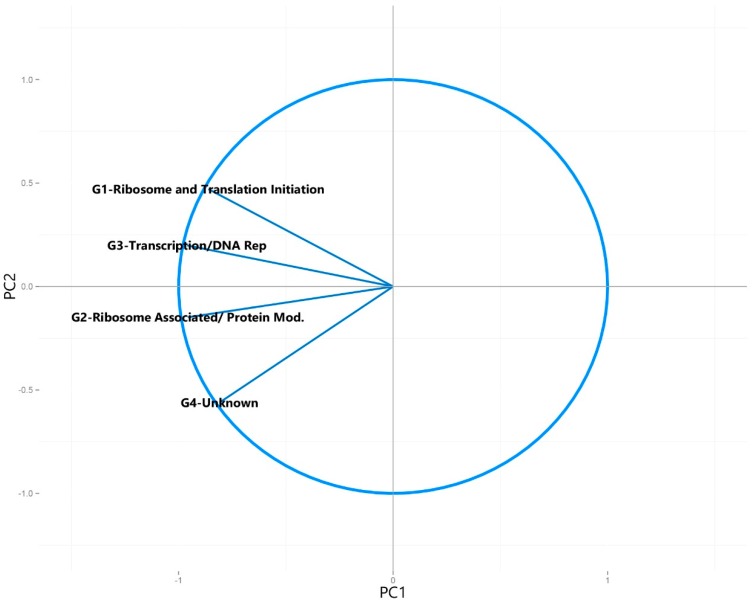
Circle of correlations generated for the principal component analysis plot of the number of universally present COGs detected in each metagenome, showing the variables that make up each of the first two principal components (Figure 2).

When principal component analysis was performed using only the totals for the universally present COGs, the resulting scree plot and statistics for each of the resulting principal components indicated that the majority of the variation (95%) was captured within the first two components (Supplemental Figure S1 and Table S3). The principal component analysis was then plotted (Figure 2) according to their previous classification (Table 1). All known and potentially syntrophic metagenomes grouped together and all non-syntrophic metagenomes grouped together with a 95% confidence ellipse (Figure 2). The variables making up the plotted principal components, as well as their direction, were also plotted in a circle of correlations (Figure 3).

The resulting plot showed no distinction between the group of the potentially syntrophic metagenomes when compared to the non-syntrophic metagenomes, with each confidence interval overlapping completely (Figure 2). In addition, the direction of the COG categories comprising the first two principal components were evenly spread, causing the metagenomes to separate left to right based on highest to lowest number of COGs in all categories, with no distinct separation in direction caused by a particular COG category (Figure 2 and Figure 3).

#### 3.2.2. Principal Component Analysis of Syntroph-Associated and Universally Present COGs

In order to determine if the abundance of syntroph associated COGs could distinguish known and potentially syntrophic metagenomes from non-syntrophic metagenomes, principal component analysis was performed using the normalized totals for the universally present COGs combined with the normalized totals for the syntroph associated COG categories (Supplemental Tables S1 and S2). 

**Figure 4:**
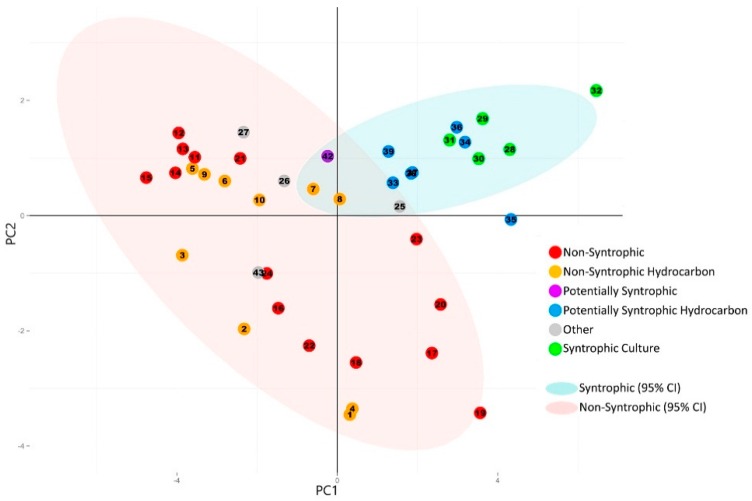
Principal component analysis plot generated from the normalized number of universally present COGs and normalized number of syntroph associated COGs detected in each metagenome. Categories for the universally present and syntroph associated COGs are listed in Table 2, with the individual COGs for each category listed in Supplemental Table S1 (universally present) and Table S2 (syntroph associated). The numbers of individual COGs found in each metagenome were summed for each COG category, divided by the total number of COGs for each respective category, and the sum was normalized against the total number of genes detected in each metagenome (Table 1 and Table 2). Principal component analysis was performed using R [18]. 95% Confidence ellipses were drawn for all metagenomes classified as syntrophic/potentially syntrophic, and for all metagenomes classified as non-syntrophic. The first two principal components are shown. Corresponding scree plot and statistics for each principal component are shown in Supplemental Figure S2 and Table S4.

**Figure 5:**
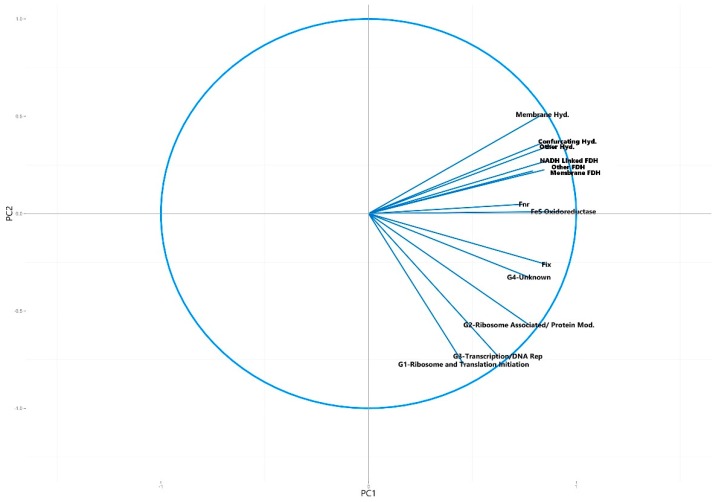
Circle of correlations for the variables in the principal component analysis plot of the number of universally present COGs and number of syntrophic gene associated COGs detected in the metagenomes, showing the contribution of each variable to the first two principal components (Figure 4). Hyd. = hydrogenase, FDH = formate dehydrogenase.

The resulting scree plot and statistics for each of the resulting principal components indicated that the majority of the variation (82%) was captured within the first two components (Supplemental Figure S2 and Table S4). The principal component analysis was then plotted according to their previous classification (Table 1). All known and potentially syntrophic metagenomes grouped together and all non-syntrophic metagenomes grouped together with a 95% confidence ellipse (Figure 4). The variables composing the first two principal components were also plotted in a circle of correlations (Figure 5).

The resulting plot showed a separation of the metagenomes with potential for syntrophy when compared to the metagenomes which were classified as non-syntrophic, with only a minimal overlap of the 95% confidence ellipses for each (Figure 4). Metagenomes that were identified as potentially syntrophic (#33–#39, #42) cluster away from this overlap, and closely together with the metagenomes from known syntrophic cultures (#28–#32). The known syntrophic culture metagenomes and ruminant metagenome previously classified in the “Other” category (#26, #27, #43) were the only samples with potentially syntrophic communities that showed clustering patterns that were different from other known syntrophic metagenomes.

When the variables making up the directions of the first two principal component axes were analyzed, it was found that the directionality of separation and clustering of the syntrophic group of metagenomes corresponded with an increasing number of syntroph associated COGs. The direction of separation caused by these COGs and therefore their contribution to the directionality of the first two principal components was distinct from that caused by the universally present COGs, indicating that these syntroph specific COGs were primarily responsible for the clustering of the syntrophic consortia into the top right quadrant of the PCA plot (Figure 4 and Figure 5). The only syntroph-associated COG category that did not contribute significantly to the separation of the syntrophic metagenomes was the “Fix” category, consisting of COGs associated with the Fix membrane-bound electron transfer flavoprotein:quinone oxidoreductases (Figure 4 and Figure 5).

This analysis was repeated using the KO gene families instead of the COG gene families for the syntroph associated genes as well as universally present genes, and a very similar clustering pattern was observed between the syntrophic and non-syntrophic metagenomes, indicating that the functional gene database used does not dramatically affect the results of the analysis (Tables S5 and S6, Figures S3–S5).

### 3.3. Comparison of Number of Syntroph-Associated COGs across Metagenomes 

**Figure 6:**
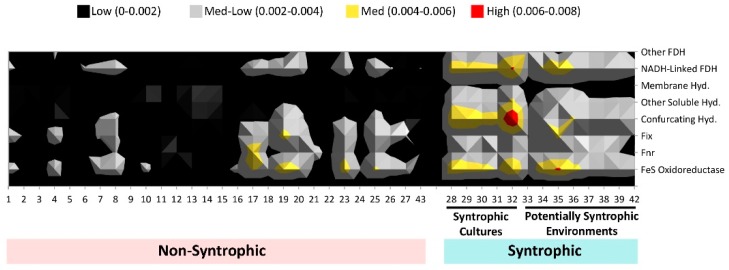
Abundance of each syntroph associated COG category in each metagenome. Numbers indicated in the legend refer to the sum total of COGs found for each category in a metagenome, normalized to the number of total genes detected in that metagenome. Individual COGs comprising each category are listed in Supplemental Table S2. The total number of COGs detected in the metagenomes were summed for each category, divided by the total number of COGs for each respective category, and normalized against the number of total genes detected in each metagenome (Table 1 and Table 2). Hyd. = hydrogenase, FDH = formate dehydrogenase.

As it appeared that the separation of the syntrophic and non-syntrophic metagenomes was driven by the abundance of the syntroph associated COGs, the sum totals for each syntroph associated COG category found in each metagenome were compared in order to determine if particular syntroph associated COGs were responsible for this trend (Figure 6). In addition, t-tests were performed on the mean numbers for each category between the non-syntrophic group and the syntrophic group in order to determine if one group had statistically higher amounts of COGs in a category than the other.

From this comparison, it was found both visually (Figure 6) as well as statistically (t-test p values for each category all < 0.05) that the largest differences between syntrophic and non-syntrophic metagenomes were in the “NADH-linked formate dehydrogenase”, “membrane bound hydrogenase”, “confurcating hydrogenase”, “other hydrogenases”, “FeS oxidoreductase”, “other formate dehydrogenase”, and “membrane formate dehydrogenase” categories, where the syntrophic metagenomes had higher mean overall COGs detected in these categories than the non-syntrophic metagenomes. The “fix” category, previously shown to not contribute to the overall separation of syntrophic metagenomes, had the highest p value for the difference of the means of the syntrophic and non-syntrophic metagenomes of all the syntroph-associated categories tested, at least 2 orders of magnitude higher than the p values for the other COG categories. Overall, the “confurcating hydrogenases” category showed the largest difference by a slight margin between mean number of COGs in this category found in metagenomes in the syntrophic and non-syntrophic metagenomes.

## 4. Discussion

Syntrophic metabolism is a key process in hydrocarbon biodegradation under methanogenic conditions, and is of central importance for both environmental remediation strategies as well as understanding the microbial potential and resource transformation in hydrocarbon resource environments. This process involves complex coordination between microorganisms in order to transfer the energy gained from substrate breakdown so that all microorganisms involved may benefit [3]. Many methanogenic hydrocarbon-degrading enrichment cultures have now been established from environmental samples and studied using a variety of approaches (e.g., reviewed in [3]). However, syntrophic partnerships that are important in environmental samples may not always be mimicked under laboratory conditions [8]. Thus, the use of metagenomics approaches (that do not involve cultivation) can offer additional insight into the species and genes involved in syntrophic hydrocarbon metabolism, as well as the energy transfer mechanisms associated with this process to allow a deeper understanding of *in situ* communities. We hypothesized that the abundance of clusters of orthologous groups (COGs) associated with key energy transfer genes prevalent in the genomes of syntrophic bacteria could be used as a tool to identify environments with syntrophic potential from metagenomic data, based on gene families associates with hydrogenase and dehydrogenase genes previously identified in pure strains as being important for syntrophy (Table 2) [8].

In order to examine the relationship between multiple syntroph-associated and universal COG groups in a variety of metagenomic sequences, principal component analysis was used. This technique helps to determine the major sources of variation within a multivariate dataset, and can separate clusters of samples based on their similarities and differences in select variables in the dataset. It is important to note that the gene count of the metagenomes used in this study was relatively comparable; using metagenomic data with gene counts that are orders of magnitude higher than the other samples due to lack of assembly or annotation can lead to the samples not being comparable after standardization of detected COG number to the total genes detected in the metagenomes. When principal component analysis was performed on the normalized number of universally present COGs found in the metagenomes, no distinction between the potentially syntrophic and non-syntrophic metagenomes could be observed (Figure 2). This is to be expected, as these COGs are universally present in almost all microbes, and therefore no separation of syntrophic and non-syntrophic groups of metagenomes should be seen once the numbers found in each metagenome are normalized for the number of genes found in the metagenome. When a circle of correlations plot was generated for this analysis, the directionality of the data spread in the metagenome plot seemed to be from the quantity of these COGs, however no single COG or group of COGs caused any particular metagenomes to separate out as a distinct cluster (Figure 2 and Figure 3). The resulting scree plot and statistics for each of the principal components calculated for this analysis indicated that the majority of the variation in the dataset (95%) was captured in the first two principal components which were plotted, indicating that the lack of separation of the two groups is not due to a component of variation in the dataset that was not plotted (Supplemental Figure S1 and Table S3).

In contrast to the analysis of the universally present COGs alone, when the normalized numbers of syntroph associated and universal COGs were combined, the principal component analysis showed separation between metagenomes classified as having syntrophic potential, and those classified as non-syntrophic (Figure 4). Metagenomes sequenced from known syntrophic laboratory consortia were found to cluster together, whether or not they were taken from consortia which degraded hydrocarbons (#28–#31) or consortia that have been reported to utilize non-hydrocarbon substrates (#32) (Figure 4) [9,10,16]. Metagenomes which were classified as potentially syntrophic, from both hydrocarbon related (#33–#39) and non-hydrocarbon related environments (#42) were found to cluster closely together with the metagenomes from known syntrophic consortia, indicating that these metagenomes all had similar traits based on the COGs used in the analysis (Figure 4) [9,10,16]. The metagenomes previously classified as belonging to the “Other” category which had originally been collected from syntrophic, methanogenic coal-degrading cultures (#26 and #27) as well as sheep rumen (#43) were found to not cluster with the other known syntrophic clusters, instead falling to the far right of the plot with the non-syntrophic metagenomes (Figure 4). Based on the microbial community composition, this could have be due to the absence of *Deltaproteobacteria* detected in these cultures, as the majority of known syntrophy-associated genes (such as the majority of those used to generate the COG set used in the analysis) are from *Deltaproteobacteria* (Figure 1, Table 2, and Supplemental Table S2) [8]. As these metagenomes were dominated by *Firmicutes* (known to be involved in syntrophic processes) and *Bacteroidetes* (found in hydrocarbon environments and syntrophic cultures, however are usually identified as primary polymer degraders and non-syntrophs), other genes involved in syntrophic energy transfer may be used in these cultures; therefore, the primarily *Deltaproteobacterial* COGs used in this analysis may differ from the COGs involved in the syntrophic cooperation present in these samples [8,24,25,26]. Because syntrophic consortia are difficult to establish, and it is even more difficult to clearly identify the genes involved in energy transfer and the overall coordination of this process, there very well may be more genes involved in energy transfer which are utilized by this culture which do not belong to the formate dehydrogenase and hydrogenase families examined in this study; for example the genes associated with DIET [11]. Alternatively, the difference in clustering pattern for the coal metagenomes may also be due to the fact that the coal metagenomes were determined using samples taken from these cultures at a relatively early point in establishment (day 16 for #26 and day 7 for #27) [16]. The syntrophic biodegradation of coal to methane is normally a very slow process, with lengthy incubation times [5,27]. Because the metagenomes of these cultures were sequenced so early-on in the incubation period, it is also possible that the full microbial community responsible for syntrophic biodegradation of coal had not been established yet, and therefore the genes (and their associated COGs) required for syntrophic energy transfer were not yet in high abundance, which could explain why these cultures did not cluster with the other known syntrophic laboratory cultures (Figure 4). In either case, the environmental metagenomes displayed a much more diverse microbial community composition than the laboratory syntrophic consortia, including increased diversity in the subset of microorganisms that could comprise a syntrophic community (Figure 1). This is to be expected, as a syntrophic community in an environmental sample would likely be more diverse than that of a laboratory culture enriched under specific conditions. Because of this, the type of sample bias that may be introduced by selecting primarily *Deltaproteobacterial* COGs when examining simple laboratory syntrophic communities such as the coal-degrading cultures does not appear to be present when examining metagenomes from environmental samples (Figure 4). The only other metagenome belonging to the “Other” category (#25) was taken from a coal bed methane deposit, where a large portion of the methane produced would have been generated from syntrophic activity [23]. Coal is rich in organic matter, and isotopic signatures in coal bed methane sites often indicate that methanogenesis is responsible for the generation of most of the methane at these locations, but this occurs over geological time, explaining the lack of a current syntrophic community or syntroph-associated genes in this sample [23].

The metagenomes classified as non-syntrophic were more spread out than the metagenomes classified as syntrophic, with separation within this category primarily driven by the number of universal COGs (Figure 4 and Figure 5). When 95% confidence ellipses were drawn around each group, it was found that the cluster of potentially syntrophic and syntrophic metagenomes (“Syntrophic”) could be seen as separate from the “Non-Syntrophic” cluster of metagenomes, with only a small area of overlap in the confidence ellipses of each group (Figure 4). This slight overlap could be expected, as many environments outside of laboratory culture would not be expected to be distinctly syntrophic or non-syntrophic; rather, a gradient between syntrophic and non-syntrophic activity would be expected.

When the variables that made up the principal components were examined using a circle of correlations plot, it was found that the separation of the syntrophic group to the top right of the plot was driven by the number of syntroph associated COGs, with metagenomes with a higher normalized number of syntroph associated COGs being placed farther to the top right than those with less of these COGs (Figure 4 and Figure 5). Of all of the syntroph associated COG categories analyzed, only the category “Fix” did not contribute to the separation of the potentially syntrophic and known syntrophic metagenomes from those which were not syntrophic (Figure 5). This category contained COGs associated with genes encoding a membrane-bound electron transfer flavoprotein:quinone oxidoreductase known as Fix, which is believed to use electrons derived from fatty acid oxidation to carry out the reduction of ferredoxin, which is unfavorable, using the energy stored in the ion gradient [8]. The Fix genes are also involved in the transfer of electrons during nitrogen fixation however, and therefore the COGs associated with these genes are likely also found in non-syntrophic environments [13]. Though this protein is likely important in regenerating reduced ferredoxin used in syntrophic energy transfer, the principal component composition indicated that the other groups of syntrophic COGs are more important in separating and identifying metagenomes with syntrophic potential [8]. The principal component analysis was also run using KO gene families as an alternative to COG families, with little difference in the end result, indicating that the gene family database used to perform the analysis does not substantially influence the results (Tables S5 and S6, Figures S3–S5).

In order to identify whether a particular COG group was more important in differentiating the syntrophic metagenomes from the non-syntrophic metagenomes, the normalized total for each syntroph-associated COG category for each metagenome was examined more closely. It was found that the largest differences in COG abundance between the known and potentially syntrophic metagenomes and the non-syntrophic metagenomes were in all categories of formate dehydrogenase and hydrogenase genes as well as FeS oxidoreductases (Figure 6). The metagenomes in the syntrophic group had more overall COGs detected in these categories than the non-syntrophic metagenomes (Figure 6). Each of these protein types is important in the generation of the small molecules (hydrogen and formate) which are often responsible for shuttling electrons in between the partner organisms involved in syntrophy, and the transfer of these two molecules is believed to be one of the main processes by which energy transfer during syntrophic biodegradation occurs [5,8].

While our analysis indicates that COG gene families can be used to detect syntrophic potential in metagenomes, it is important to note that many of these gene families are still poorly understood in metagenomes, especially those outside of medical studies. In addition, certain COG categories (such as the FeS oxidoreductases) are not well defined and annotated in metagenomes, and encompass a large amount of different proteins with diverse functions. Thus, work remains to be done in better refining and annotating these categories in metagenomic datasets, so that the functional genes that they represent can be narrowed in scope to be more specific for predicting functions and relationships from metagenomic data. With this in mind, the analysis conducted here shows that even with this lack of specificity, the presence of the syntroph-associated COGs at a metagenomic scale may be used to indicate the potential for syntrophic metabolism in a given metagenome. 

## 5. Conclusions

Overall, it was found that examining key clusters of orthologous groups (COGs) related to syntrophic energy transfer genes present in different metagenome sequences using principal component analysis was able to distinguish environments with syntrophic potential from those which were non-syntrophic. Predominant differences appeared to arise from an increased number of COGs related to hydrogenase and formate dehydrogenase proteins between syntrophic and non-syntrophic metagenomes. The outcome of the analysis provides evidence that hydrogenase and dehydrogenase enzymes, postulated as being key to energy transfer reactions in syntrophic co-cultures, extend to mixed syntrophic communities that characterize many environments. This kind of analysis could be used in the future in order to assess the syntrophic potential of other environmental metagenomes as they become available, as well as to identify other COGs that are correlated with and potentially involved in syntrophy. 

## Supplementary Materials

Supplementary materials can be found at www.mdpi.com/2076-2607/4/1/5/s1.
