# Supplementary material for: Metagenomic Analyses Reveal That Energy Transfer Gene Abundances Can Predict the Syntrophic Potential of Environmental Microbial Communities
Source: Microorganisms. 2016 Jan 5;4(1):5. doi: 10.3390/microorganisms4010005 (PMC5029510; doi:10.3390/microorganisms4010005)
Supplement: Supplementary file 1 [file microorganisms-04-00005-s001.docx]

**Supplementary Materials: Metagenomic Analyses Reveal That Energy Transfer Gene Abundances Can Predict the Syntrophic Potential of Environmental Microbial Communities**

Lisa Oberding and Lisa Gieg

**Table S1.** Universally present COGs. COGs were obtained from published literature, and COGs associated with genes in each category were obtained using the IMG database [1,2].

| **G1-Ribosome and Translation Initiation** | **G2-Ribosome Associated/ Protein Modification** | **G3-Transcription/DNA Replication** | **G4-Unknown** |
| --- | --- | --- | --- |
| Function ID | Function ID | Function ID | Function ID |
| COG0048 | COG0006 | COG0085 | COG0012 |
| COG0049 | COG0024 | COG0086 | COG0037 |
| COG0050 | COG0112 | COG0180 |  |
| COG0051 | COG0201 | COG0202 |  |
| COG0052 | COG0541 | COG0250 |  |
| COG0080 | COG0552 | COG0258 |  |
| COG0081 |  | COG0468 |  |
| COG0087 |  | COG0592 |  |
| COG0088 |  |  |  |
| COG0089 |  |  |  |
| COG0090 |  |  |  |
| COG0091 |  |  |  |
| COG0093 |  |  |  |
| COG0094 |  |  |  |
| COG0096 |  |  |  |
| COG0097 |  |  |  |
| COG0098 |  |  |  |
| COG0099 |  |  |  |
| COG0100 |  |  |  |
| COG0102 |  |  |  |
| COG0103 |  |  |  |
| COG0184 |  |  |  |
| COG0185 |  |  |  |
| COG0186 |  |  |  |
| COG0197 |  |  |  |
| COG0198 |  |  |  |
| COG0199 |  |  |  |
| COG0231 |  |  |  |
| COG0244 |  |  |  |
| COG0256 |  |  |  |
| COG0361 |  |  |  |
| COG0480 |  |  |  |
| COG0522 |  |  |  |
| COG0532 |  |  |  |

**Table S2.** COGs associated with syntroph-associated energy transfer gene categories. Gene sequences were obtained from published literature, and COGs associated with genes in each category were obtained using the IMG database [2,3]. Hyd. = hydrogenase, FDH = formate dehydrogenase.

| **FeS Oxidoreductase** | **Fnr** | **Fix** | **Confurcating Hyd.** | **Other Hyd.** | **Membrane Hyd.** | **NADH Linked FDH** | **Other FDH** | **Membrane FDH** |
| --- | --- | --- | --- | --- | --- | --- | --- | --- |
| Function  ID | Function ID | Function ID | Function  ID | Function ID | Function ID | Function ID | Function ID | Function ID |
| COG0247 | COG0001 | COG0644 | COG1034 | COG0375 | COG0374 | COG0303 | COG0437 | COG0243 |
| COG2025 | COG0577 | COG2025 | COG1143 | COG0437 | COG0437 | COG0493 | COG5557 | COG0437 |
| COG2086 | COG1136 | COG2086 | COG1894 | COG0543 | COG0650 | COG0564 |  | COG0746 |
|  | COG2878 | COG2440 | COG1905 | COG0650 | COG0651 | COG0565 |  | COG2864 |
|  | COG4656 |  | COG3411 | COG0651 | COG1006 | COG1541 |  | COG5557 |
|  | COG4657 |  | COG4231 | COG0680 | COG1320 | COG1894 |  |  |
|  | COG4658 |  | COG4624 | COG0852 | COG1563 | COG1905 |  |  |
|  | COG4659 |  |  | COG1009 | COG1740 | COG2116 |  |  |
|  | COG4660 |  |  | COG1034 | COG1863 | COG3411 |  |  |
|  |  |  |  | COG1035 | COG1894 |  |  |  |
|  |  |  |  | COG1142 | COG1905 |  |  |  |
|  |  |  |  | COG1143 | COG1908 |  |  |  |
|  |  |  |  | COG1148 | COG1941 |  |  |  |
|  |  |  |  | COG1171 | COG1969 |  |  |  |
|  |  |  |  | COG1894 | COG2111 |  |  |  |
|  |  |  |  | COG1905 | COG2212 |  |  |  |
|  |  |  |  | COG1908 | COG2864 |  |  |  |
|  |  |  |  | COG1941 | COG3259 |  |  |  |
|  |  |  |  | COG3259 | COG3260 |  |  |  |
|  |  |  |  | COG3260 | COG3261 |  |  |  |
|  |  |  |  | COG3261 | COG3262 |  |  |  |
|  |  |  |  | COG4624 | COG3411 |  |  |  |
|  |  |  |  | COG4656 | COG4624 |  |  |  |
|  |  |  |  |  | COG5557 |  |  |  |

**Table S3.** Statistics of the scaled principal component analysis performed on the number of universally present COGs detected in the metagenomes. Numbers of detected COGs were summed for each category, divided by the total number of COGs in each respective category, and normalized using the total number of genes in each metagenome before processing with principal component analysis in R [4].

|  | **PC1** | **PC2** | **PC3** | **PC4** |
| --- | --- | --- | --- | --- |
| Standard Deviation | 1.7988 | 0.7732 | 0.33565 | 0.23159 |
| Proportion of Variance | 0.8089 | 0.1495 | 0.02816 | 0.01341 |
| Cumulative Proportion | 0.8089 | 0.9584 | 0.98659 | 1.00000 |

**Table S4.** Statistics of the scaled principal component analysis performed on the number of universally present COGs and the number of syntrophic gene associated COGs detected in the metagenomes. Principal components 6 to 13 are not shown. Numbers of detected COGs were summed for each category, divided by the total number of COGs in each respective category, and normalized using the total number of genes in each metagenome before processing with principal component analysis in R [4].

|  | **PC1** | **PC2** | **PC3** | **PC4** | **PC5** |
| --- | --- | --- | --- | --- | --- |
| Standard Deviation | 2.8922 | 1.5306 | 0.86711 | 0.70585 | 0.61028 |
| Proportion of Variance | 0.6434 | 0.1802 | 0.05784 | 0.03833 | 0.02865 |
| Cumulative Proportion | 0.6434 | 0.8237 | 0.88150 | 0.91982 | 0.94847 |

**Table S5.** KOs for gene categories. Gene sequences were obtained from published literature, and KOs associated with genes in each category were obtained using the IMG database [2,3]. Universal KOs were obtained from literature [5]. Hyd= hydrogenase, FDH= formate dehydrogenase.

| **FeS Oxidored.** | **Fnr** | **Fix** | **Confurcating Hyd.** | **Other Hyd.** | **Membrane H_2_ase** | **NADH Linked FDH** | **Other FDH** | **Membrane FDH** | **Universal** |
| --- | --- | --- | --- | --- | --- | --- | --- | --- | --- |
| Function ID | Function ID | Function ID | Function ID | Function ID | Function ID | Function ID | Function ID | Function ID | Function ID |
| K03521 | K01845 | K00313 | K00123 | K00196 | K00124 | K00123 | K00123 | K00123 | K00133 |
| K03522 | K02003 | K03521 | K00334 | K00334 | K00127 | K00334 | K00124 | K00124 | K00789 |
|  | K02004 | K03522 | K00335 | K00335 | K00437 | K00335 |  | K00127 | K00927 |
|  | K03612 | K03855 | K00336 | K00336 | K00532 | K01912 |  | K02379 | K00939 |
|  | K03613 |  | K17992 | K00337 | K00533 | K02379 |  | K02380 | K01689 |
|  | K03614 |  | K18330 | K00436 | K00534 | K02533 |  | K03116 | K01803 |
|  | K03615 |  | K18331 | K00441 | K03620 | K03750 |  |  | K01866 |
|  | K03616 |  | K18332 | K00442 | K05566 | K05299 |  |  | K01867 |
|  | K03617 |  |  | K01751 | K05567 | K06180 |  |  | K01868 |
|  |  |  |  | K03388 | K05568 | K08177 |  |  | K01869 |
|  |  |  |  | K04651 | K05569 | K09793 |  |  | K01870 |
|  |  |  |  | K05586 | K05570 | K17992 |  |  | K01872 |
|  |  |  |  | K05587 | K05571 | K18330 |  |  | K01873 |
|  |  |  |  | K05588 | K06281 | K18331 |  |  | K01874 |
|  |  |  |  | K14086 | K06282 |  |  |  | K01875 |
|  |  |  |  | K14087 | K06441 |  |  |  | K01876 |
|  |  |  |  | K14088 | K14126 |  |  |  | K01881 |
|  |  |  |  | K14089 | K14127 |  |  |  | K01883 |
|  |  |  |  | K14090 | K14128 |  |  |  | K01887 |
|  |  |  |  | K14091 | K17992 |  |  |  | K01889 |
|  |  |  |  | K14126 | K18008 |  |  |  | K01890 |
|  |  |  |  | K14127 | K18016 |  |  |  | K01892 |
|  |  |  |  | K14128 | K18017 |  |  |  | K01937 |
|  |  |  |  | K17996 | K18023 |  |  |  | K02357 |
|  |  |  |  | K18007 | K18330 |  |  |  | K02519 |
|  |  |  |  |  | K18331 |  |  |  | K02528 |
|  |  |  |  |  |  |  |  |  | K02600 |

**Table S6.** Statistics of the scaled principal component analysis performed on the number of universally present KOs and the number of syntrophic gene associated KOs detected in the metagenomes. Principal components 6 to 13 are not shown. Numbers of detected KOs were summed for each category, divided by the total number of KOs in each respective category, and normalized using the total number of genes in each metagenome before processing with principal component analysis in R [4].

|  | **PC1** | **PC2** | **PC3** | **PC4** | **PC5** |
| --- | --- | --- | --- | --- | --- |
| Standard Deviation | 2.7053 | 0.98391 | 0.78111 | 0.66211 | 0.50432 |
| Proportion of Variance | 0.7318 | 0.09681 | 0.06101 | 0.04384 | 0.02543 |
| Cumulative Proportion | 0.7318 | 0.82865 | 0.88966 | 0.93350 | 0.95893 |


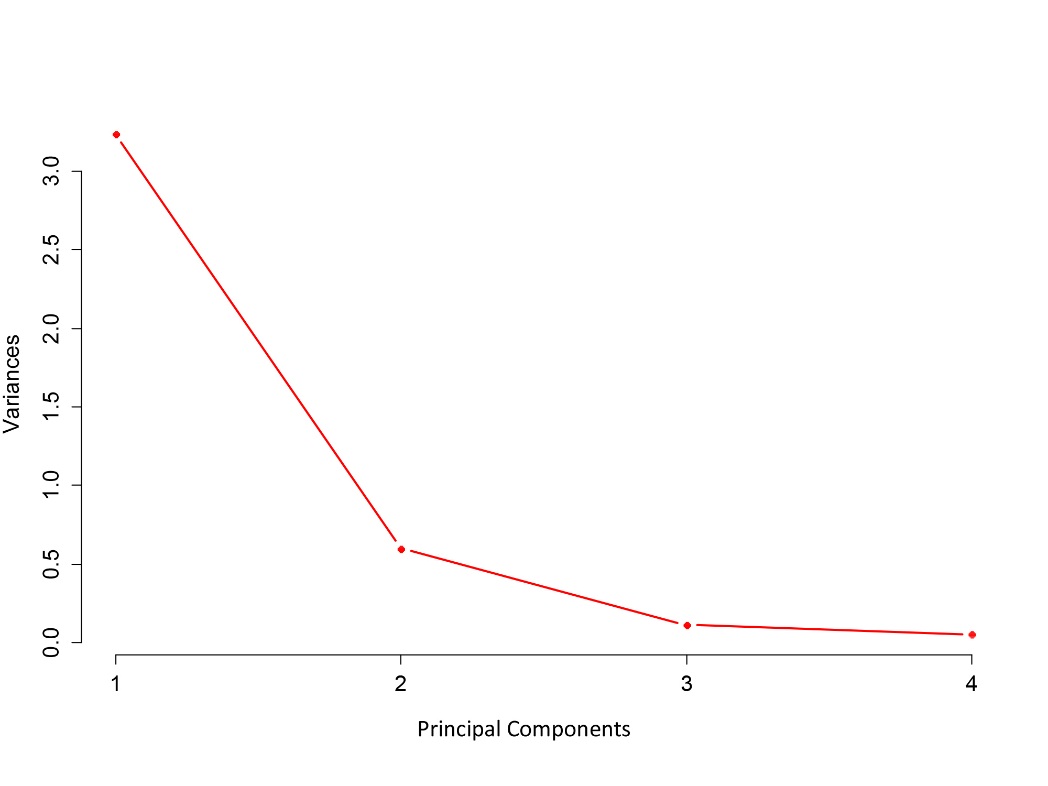


**Figure S1.** Scree plot of the scaled principal component analysis performed on the number of universally present COGs detected in the metagenomes. Numbers of detected COGs were summed for each category, divided by the total number of COGs in each respective category, and normalized using the total number of genes in each metagenome before processing with principal component analysis in R [4].


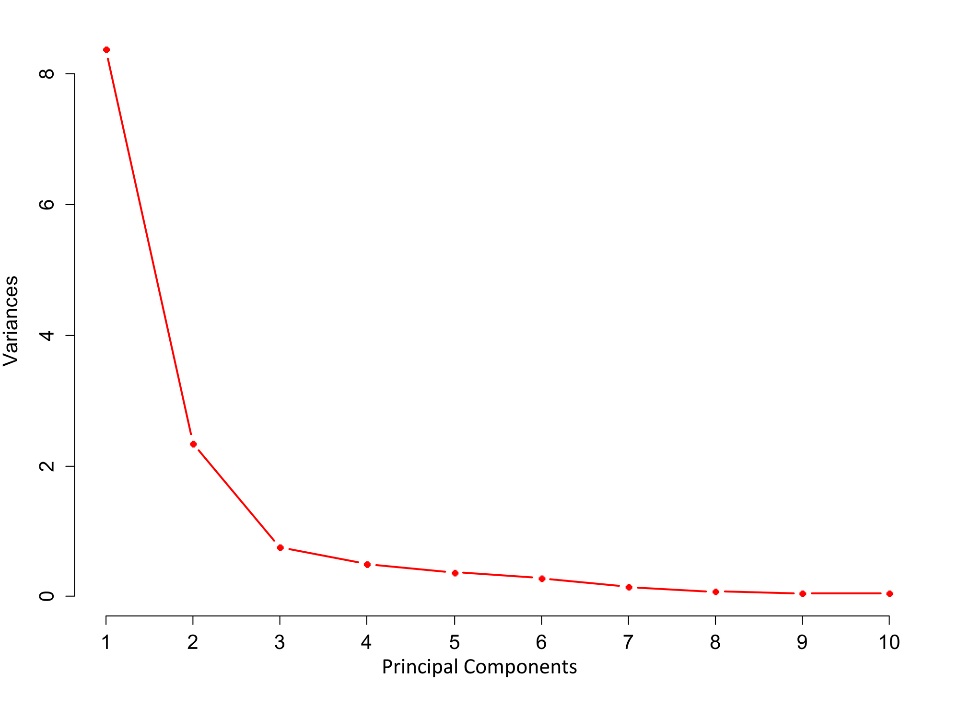


**Figure S2.** Scree plot of the scaled principal component analysis performed on the number of universally present COGs and the number of syntrophic gene associated COGs detected in the metagenomes. Numbers of detected COGs were summed for each category, divided by the total number of COGs in each respective category, and normalized using the total number of genes in each metagenome before processing with principal component analysis in R [4].


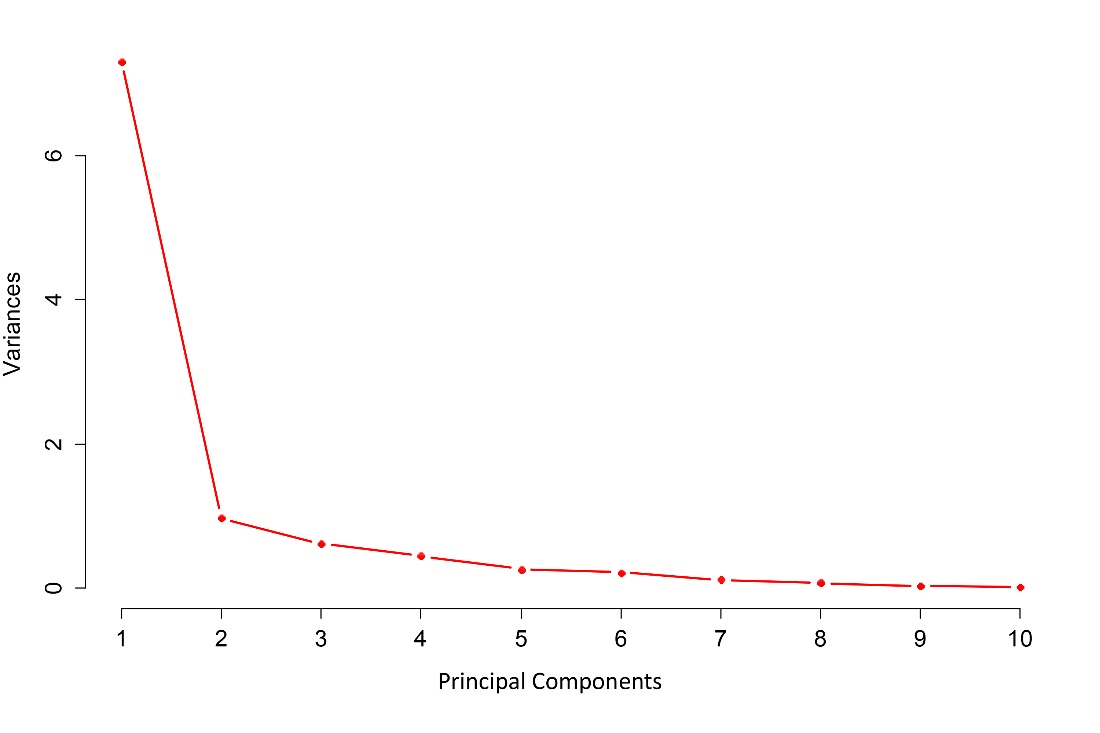


**Figure S3.** Scree plot of the scaled principal component analysis performed on the number of universally present KOs and the number of syntrophic gene associated KOs detected in the metagenomes. Numbers of detected KOs were summed for each category, divided by the total number of KOs in each respective category, and normalized using the total number of genes in each metagenome before processing with principal component analysis in R [4].


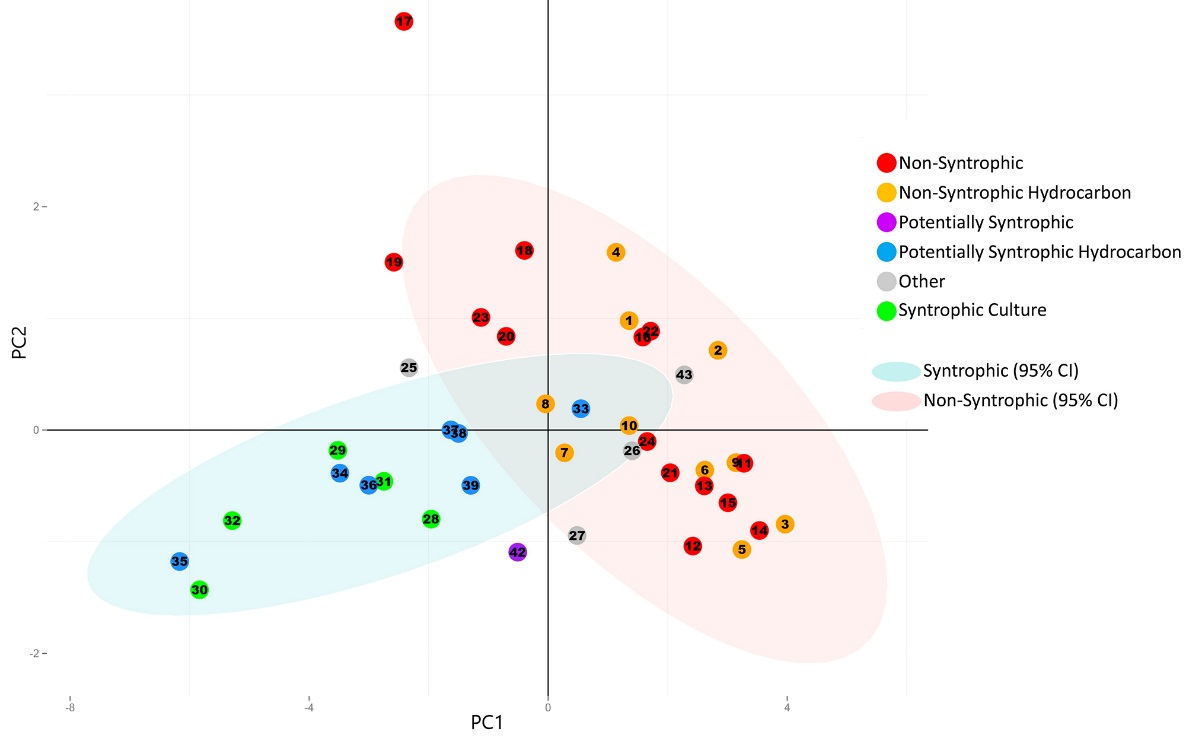


**Figure S4.** Scree plot of the scaled principal component analysis performed on the number of universally present KOs and the number of syntrophic gene associated KOs detected in the metagenomes. Numbers of detected KOs were summed for each category, divided by the total number of KOs in each respective category, and normalized using the total number of genes in each metagenome before processing with principal component analysis in R [4].


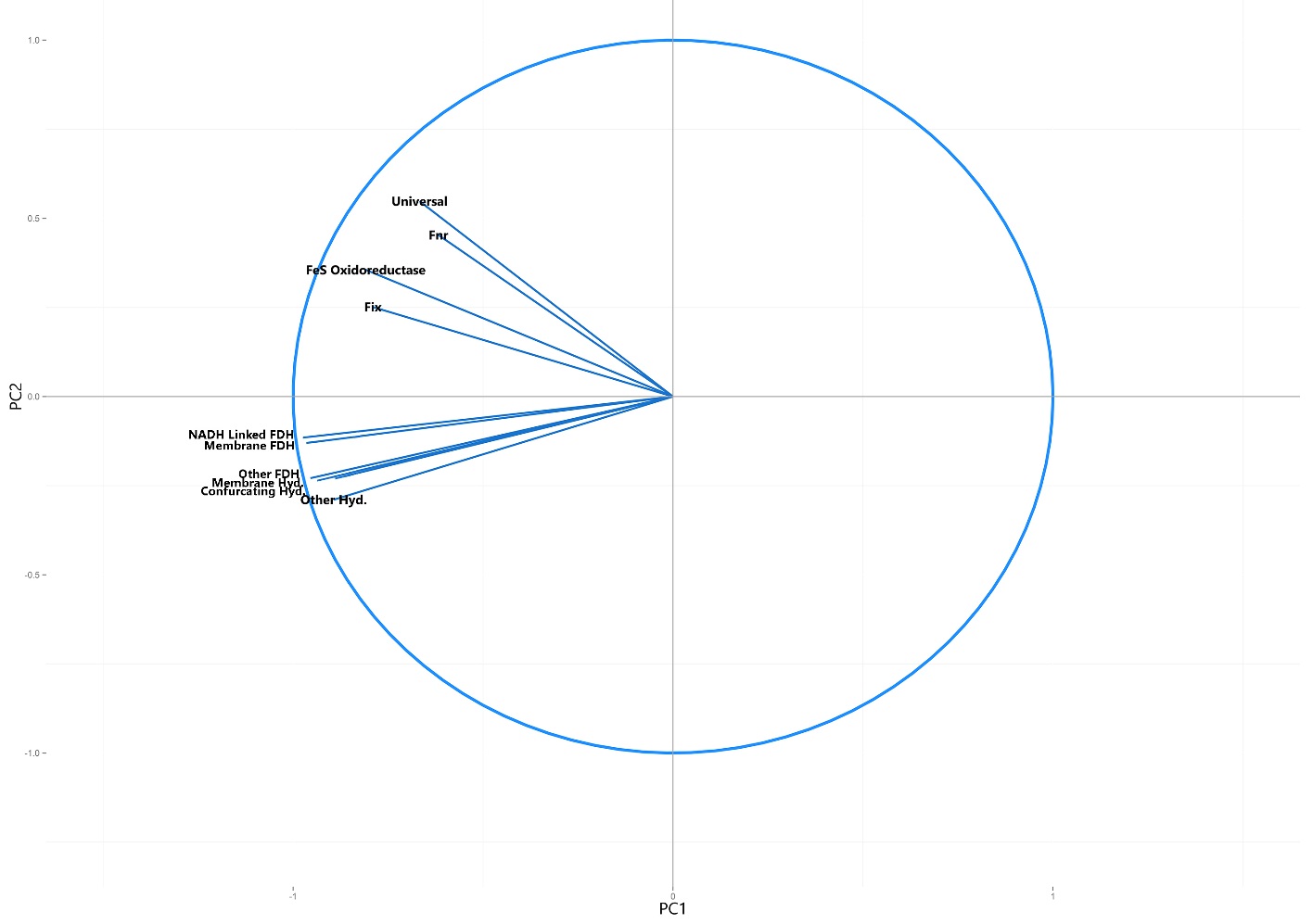


**Figure S5.** Circle of correlations generated for the principal component analysis plot of the number of universally present KOs and syntroph associated KOs detected in each metagenome, showing the variables that make up each of the first two principal components (Figure S4).

R Script to support the article:

#NOTE: Import one dataset and run full analysis to generate all figures for that data set.

#NOTE: If a library isnt present, use install.packages(NAMEOFLIBRARY) to install first, then run the library() script

#To import data (Universal and Syntroph Associated Normalized Totals Per Metagenome)

metagenomes<-read.csv("School//Graduate//Grad Work/Experiments/Syntrophy Metagenomics/R Data CSV/UniversalAndSieberCOG.csv",header=FALSE,sep=",")

#Set the column names for each of the gene categories analyzed

colnames(metagenomes)<-c("G1-Ribosome and Translation Initiation","G2-Ribosome Associated/ Protein Mod.","G3-Transcription/DNA Rep","G4-Unknown","FeS Oxidoreductase","Fnr","Fix","Confurcating H2ase","Other H2ase","Membrane H2ase","NADH Linked FDH","Other FDH","Membrane FDH")

#Set the rownames to the numbers in the table (data must already be in the order that the table is in)

rownames(metagenomes)=c(1:43)

#To import data (Universal Normalized Totals Per Metagenome)

metagenomes<-read.csv("School//Graduate//Grad Work/Experiments/Syntrophy Metagenomics/R Data CSV/FullListCOGUniversal.csv",header=FALSE,sep=",")

colnames(metagenomes)<-c("G1-Ribosome and Translation Initiation","G2-Ribosome Associated/ Protein Mod.","G3-Transcription/DNA Rep","G4-Unknown")

rownames(metagenomes)=c(1:43)

#To display imported dataset

metagenomes

#To remove metagenomes 40 and 41 in my dataset, as they are not part of this analysis

metagenomes<-metagenomes[-40:-41,]

#to perform principal component analysis on dataset, setting scale=T to scale data

pca1<-prcomp(metagenomes,scale=T)

#to store rotated data scores in order to plot later

scores=as.data.frame(pca1$x)

#-------------------------------------------

#To plot the scree plot of the pca analysis

library(ggplot2)

library(extrafont)

plot(pca1, type="l", col="red", lwd=2, pch=16)

plot(pca1, type="b", col="black", lwd=2, pch=16)

#To obtain the scores of the principal components and the variation captured by each

summary(pca1)

#############################################################

#PCA Plot + 95%CI#

#############################################################

#define the category for each metagenome

treat=c("Non-Syntrophic Hydrocarbon","Non-Syntrophic Hydrocarbon","Non-Syntrophic Hydrocarbon","Non-Syntrophic Hydrocarbon","Non-Syntrophic Hydrocarbon","Non-Syntrophic Hydrocarbon","Non-Syntrophic Hydrocarbon","Non-Syntrophic Hydrocarbon","Non-Syntrophic Hydrocarbon","Non-Syntrophic Hydrocarbon",

"Non-Syntrophic","Non-Syntrophic","Non-Syntrophic","Non-Syntrophic","Non-Syntrophic","Non-Syntrophic","Non-Syntrophic","Non-Syntrophic","Non-Syntrophic","Non-Syntrophic","Non-Syntrophic","Non-Syntrophic","Non-Syntrophic","Non-Syntrophic",

"Other","Other","Other",

"Syntrophic Consortia","Syntrophic Consortia","Syntrophic Consortia","Syntrophic Consortia","Syntrophic Consortia",

"Potentially Syntrophic Hydrocarbon","Potentially Syntrophic Hydrocarbon","Potentially Syntrophic Hydrocarbon","Potentially Syntrophic Hydrocarbon","Potentially Syntrophic Hydrocarbon","Potentially Syntrophic Hydrocarbon","Potentially Syntrophic Hydrocarbon"

)

,"Potentially Syntrophic","Other")

#define the overall syntrophic or non-syntrophic groups

a=c(rep("No",27))

b=c(rep("Yes",12))

c=c("Yes","No")

syn=c(a,b,c)

#plot the PCA

library(devtools)

library(digest)

myplot=ggplot(data = scores, aes(x = PC1, y = PC2, color=syn), alpha=0.5) +

#plot the 95% CI for the syntrophic and nonsyntrophic groups

stat_ellipse(aes(x=PC1,y=PC2, fill=factor(syn)),geom="polygon", level=0.95, alpha=0.1)+

#to change graph axis= coord_fixed(xlim = c(-5,5),ylim = c(-4.5,4.5)) +

geom_hline(yintercept = 0, colour = "black") +

geom_vline(xintercept = 0, colour = "black") +

#This colors the point by the individual genome- can color by (treat) if wanted

#geom_point(aes(color = factor(1:nrow(metagenomes))), lwd=8)+

geom_point(aes(color = factor(treat)), lwd=8)+

geom_text(size=4, face="bold", family="Arial Black", color="black", label=rownames(scores))+ #hjust=0, vjust=0) +

ggtitle("PCA plot of Metagenomes")+

theme(axis.title=element_text(family="Segoe UI",size="18", color="black"))+

theme(plot.title=element_text(family="Segoe UI",size="20", face="bold", color="black"))

myplot + theme(panel.background = element_blank())

#Color the metagenomes by group

myplot<-myplot+ element_line(scale_colour_manual(values=c("white","red","orange","grey","purple","dodgerblue","green","white")))

myplot=myplot + theme(panel.background = element_blank())

plot(myplot)

#############################################################

#Circle of Correlations#

#############################################################

circle <- function(center = c(0, 0), npoints = 100) {

r = 1

tt = seq(0, 2 * pi, length = npoints)

xx = center[1] + r * cos(tt)

yy = center[1] + r * sin(tt)

return(data.frame(x = xx, y = yy))

}

corcir = circle(c(0, 0), npoints = 100)

correlations = as.data.frame(cor(metagenomes, pca1$x))

#If the code below breaks, try changing one or the other or both ncol to nrow, or nrow to ncol (try all four variations)

arrows = data.frame(x1 = c(rep(0,nrow(correlations))), y1 = c(rep(0,ncol(correlations))), x2 = correlations$PC1,

y2 = correlations$PC2)

circleplot<-ggplot() + geom_path(data = corcir, aes(x = x, y = y), colour = "dodgerblue", lty=1, lwd=1.5) +

geom_segment(data = arrows, aes(x = x1, y = y1, xend = x2, yend = y2), colour = "dodgerblue3", lwd=1) +

geom_text(data = correlations, color="black", aes(x = PC1, y = PC2, label = rownames(correlations),family="Segoe UI"), size=5, fontface="bold") +

geom_hline(yintercept = 0, colour = "gray65") +

geom_vline(xintercept = 0, colour = "gray65") +

xlim(-1.5, 1.5) + ylim(-1.25, 1.25) + labs(x = "PC1", y = "PC2", family="Segoe UI") +

ggtitle("Circle of Correlations")+

theme(axis.title=element_text(family="Segoe UI",size="18", color="black"))+

theme(plot.title=element_text(family="Segoe UI",size="20", face="bold", color="black"))

circleplot=circleplot + theme(panel.background = element_blank())

circleplot

References

1. Harris, J.K.; Kelley, S.T.; Spiegelman, G.B.; Pace, N.R. The genetic core of the universal ancestor. *Genome Res.* **2003**, *13*, 407–412.
2. Markowitz, V.M.; Chen, I.M.A.; Palaniappan, K.; Chu, K.; Szeto, E.; Pillay, M.; Ratner, A.; Huang, J.; Woyke, T.; Huntemann, M.; *et al*. IMG 4 version of the integrated microbial genomes comparative analysis system. *Nucleic Acids Res*. **2014**, *42*, D560–D567.
3. Sieber, J.R.; McInerney, M.J.; Gunsalus, R.P. Genomic insights into syntrophy: The paradigm for anaerobic metabolic cooperation. *Annu. Rev. Microbiol*. **2012**, *66*, 429–452.
4. R Development Core Team. R: A language and environment for statistical computing. Available online: https://www.r-project.org (accessed on 29 October 2015).
5. Manor, O.; Borenstein, E. MUSiCC: A marker genes based framework for metagenomic normalization and accurate profiling of gene abundances in the microbiome. *Genome Biol.* **2015**, doi:10.1186/s13059-015-0610-8.
